# Enolase-like protein present on the outer membrane of *Pseudomonas aeruginosa* binds plasminogen

**DOI:** 10.1007/s12223-014-0311-9

**Published:** 2014-03-27

**Authors:** Ireneusz Ceremuga, Ewa Seweryn, Iwona Bednarz-Misa, Jadwiga Pietkiewicz, Katarzyna Jermakow, Teresa Banaś, Andrzej Gamian

**Affiliations:** 1 Department of Medical Biochemistry, Wroclaw Medical University, Chalubinskiego 10, 50-368 Wroclaw, Poland; 2 Department of Microbiology, Wroclaw Medical University, Chalubinskiego 4, 50-368 Wroclaw, Poland

## Abstract

*Pseudomonas aeruginosa* is one of the pathogenic bacteria which utilize binding of the host plasminogen (Plg) to promote their invasion throughout the host tissues. In the present study, we confirmed that *P. aeruginosa* exhibits binding affinity for human plasminogen. Furthermore, we showed that the protein detected on the cell wall of *P. aeruginosa* and binding human plasminogen is an enolase-like protein. The hypothesis that alpha-enolase, a cytoplasmatic glycolytic enzyme, resides also on the cell surface of the bacterium was supported by electron microscopy analysis. The plasminogen-binding activity of bacterial cell wall outer membrane enolase-like protein was examined by immunoblotting assay.

## Introduction

*Pseudomonas aeruginosa*, a widespread opportunistic pathogen, belongs to the Gram-negative non-fermenting bacteria of *Pseudomonadaceae* family. It is an etiologic agent of several respiratory, urinary, and gastrointestinal infections. Out of several known factors determining its pathogenicity, the exopolysaccharide mucus (glycocalyx) seems to be of greatest importance. It plays a prime role in cellular adhesion and persistent bacterial colonization of the epithelium and other cells. Furthermore, it exhibits immunogenic properties. *P. aeruginosa* secretes a number of cytotoxic exoenzymes, such as phospholipid-cleaving phospholipase C and proteases degrading the host’s proteins (Lomholt et al. 2001). The bacterium is particularly dangerous for immunocompromised individuals. Scientific reports suggest *P. aeruginosa* as the most important pathogen and causal agent of chronic pulmonary infections in patients with cystic fibrosis, which leads to gradual and eventually fatal decline in pulmonary function (Govan and Deretic 1996). *P. aeruginosa* shows low susceptibility to many classes of antibiotics (Hancock and Speert 2000; Jo et al. 2003). Immunoprophylaxis and immunotherapy (vaccines for the immunocompromised and passive immunization with hyperimmune sera) are the most promising and therefore of highest interest. Over many years, efforts to produce effective vaccines against *P. aeruginosa* were focused on two factors playing an important role in the bacterial pathogenicity: carbohydrate structures represented by lipopolysaccharides and polysaccharides of the bacterial cellular capsules and proteinaceous components of their external membranes (Campbell et al. 1996; Kim et al. 2000). Vaccines based on the latter proved safer due to higher tolerance by humans (Larbig et al. 2001; Doring and Pier 2008). The process of host colonization and further development of infection rely on the proteins of the bacterial external cellular membrane. The proteins interacting with the host’s tissues initiate the degradation of physiological functions of the invaded organism, stimulate the progression of infection, and facilitate adaptation to the external environment (Lang 2000; Lin et al. 2002). The proteins exposed on the cellular membrane exhibit similar characteristics; however, it remains unclear whether they determine the course of infection. Most probably, these proteins possess auxiliary function. Enolase is a glycolytic enzyme present in eukaryotes and prokaryotes (Seweryn et al. 2007, 2009). In humans, besides catalyzing glycolysis and gluconeogenesis, it composes membranes of epithelial and endothelial cells, monocytes, leucocytes, and neutrophiles where it acts as a receptor of human plasminogen (Pancholi and Fischetti 1998; Pancholi 2001; Lopez-Alemany et al. 2003; Diaz-Ramos et al. 2012). Plasminogen plays a crucial role in maintaining coagulation homeostasis. Converted into active plasmin, which in turn degrades fibrin, it allows the white blood cells to migrate to the site of inflammation. The presence of protein receptors of plasminogen on the cellular surface enables the invasive bacteria to employ the fibrinolytic activity of plasmin and thus cleave the laminin, fibronectin, proteoglycans, and other components of human extracellular matrices (Kinloch et al. 2005; Agarwal et al. 2008). The ability to transmigrate through the layers of pulmonary epithelium and vascular endothelium allows the bacteria to breach the tissue barrier and colonize the host’s tissues.

## Materials and methods

### Obtaining and purification of the enolase-like surface protein from *P. aeruginosa* cells

*P. aeruginosa* cells were cultured on tryptic soy agar (TSA) solid medium for 48 h at the temperature of 37 °C. The bacterial mass (18.7 g) was washed with pH 7.2, 10 mmol/L Tris-HCl buffer with 3 mmol/L MgSO_4_, 1 % glycerol, and 0.5 mmol/L beta-mercaptoethanol and suspended in three volumes of the same buffer containing also 4 μg/mL phenylmethylsulfonyl fluoride (PMSF) and 0.2 μL/mL aprotinin and was frozen in −80 °C. The purity of the culture was assessed by microscopy and on agar plates. The separation of cellular structures was achieved with two freeze/thaw cycles at −80 °C and room temperature. The sonicated material was suspended in the following buffer: 10 mmol/L Tris-Cl, pH 7.2, with 1 % glycerol, 3 mmol/L MgSO_4_, 0.5 mmol/L beta-mercaptoethanol, 4 μg/mL PMSF, and 0.2 μL/mL aprotinin. Undisrupted cells were removed by centrifugation at 4,000 *g*, for 40 min at 4 °C. The resulting supernatant was centrifuged at 120,000 *g* for 1 h at 4 °C in order to separate the membrane fraction (precipitate) from the cytosolic one (supernatant). The precipitate containing the membrane fraction (external and cytoplasmic membranes) was extracted twice in the following buffer A: 10 mmol/L Tris-Cl, pH 7.2, with 1 % glycerol, 3 mmol/L MgSO_4_, 1 mmol/L beta-mercaptoethanol, and 2 % Triton X-100, for 30 min at 20 °C and centrifuged (120,000 *g* for 1 h at 4 °C). Next, in order to rinse off proteins of external membranes, the precipitate was extracted twice with buffer A containing 5 mmol/L EDTA (buffer B) for 30 min at 20 °C and centrifuged (120,000 *g* for 1 h at 4 °C). The obtained supernatants from both extractions were combined in buffer B and dialyzed into the following buffer: 10 mmol/L Tris-Cl, pH 7.0, with 3 mmol/L MgSO_4_ and 1 mmol/L beta-mercaptoethanol, and subsequently concentrated by lyophilization. The obtained membrane proteins were incubated once for 5 min at 100 °C with a reducing buffer (50 mmol/L Tris-HCl (pH 6.8), 20 % glycerol, 5 % sodium dodecyl sulfate (SDS), 0.005 % bromophenol blue, 10 % beta-mercaptoethanol) in the proportion 1:5 and separated with the Prep-Cell 491 device (Bio-Rad) in polyacrylamide gel gradient: 50 mL of 10 % and 35 mL of 12 % separating gel with 20 mL of 4 % condensing gel. The proteins were separated at 250 V and 4 °C in an electrode buffer: 25 mmol/L Tris-HCl and 0.192 mol/L glycine, pH 8.3, with 0.5 % SDS. Protein elution with the electrode buffer was started once the bromophenol blue reached the basis of the separating gel. Collected at the rate of 0.75 mL/min, 1.3-mL fractions were subsequently poured into deionized water and concentrated by lyophilization.

### Immunoblotting identification of the enolase-like protein

The bacterial enolase-like protein was detected with rabbit anti-human alpha-enolase antibodies obtained in our laboratory (data not shown). Once separated in polyacrylamide gel, the proteins were transferred to the Immobilon P membrane. The transfer followed Witkowska’s guidelines (Witkowska et al. 2005) in a transfer buffer (10 mmol/L Tris, 150 mmol/L glycine, 20 % methanol) for 120 min at 200 mA. After the transfer, the membrane was rinsed with water thrice in order to remove buffer traces and stained with 0.005 % Ponceau S in 3 % trichloroacetate with the aim to assess the quality of protein separation and transfer. The membrane was washed with deionized water. The membranes containing transferred proteins were incubated at 37 °C for 1 h with rabbit anti-human alpha-enolase antibodies diluted 1:1,000 in TBS-T (20 mmol/L Tris-HCl, pH 7.0, with 50 mmol/L NaCl and 0.05 % Tween-20) with 1 % bovine serum albumin (BSA). Unbound antibodies were washed off with TBS (20 mmol/L Tris-HCl, pH 7.0, with 50 mmol/L NaCl). Next, the membrane was incubated at 37 °C for 1 h with goat anti-rabbit IgG antibodies coupled with peroxidase.

### Evaluation of enzymatic activity of surface proteins of *P. aeruginosa*

Surface proteins of undisrupted cells were evaluated for enolase-specific enzymatic activity as described by Pancholi (Pancholi and Fischetti 1998). The evaluation was made on bacterial cells in the phase of exponential growth. The bacteria (counted according to McFarland’s scale concentration, 1.95 × 10^10^ per milliliter) were washed thrice with a reaction buffer (100 mmol/L HEPES/NaOH, pH 7.2, with 10 mmol/L MgSO_4_ and 7.7 mmol/L KCl) and suspended in different concentrations in 10 mL of reactive buffer. Enolase substrate 2-phosphoglycerate (2-PGA) was added to the samples at 6-mmol/L concentration. For control, the bacteria were incubated under identical conditions without the substrate. The product accumulation phosphoenolpyruvate (PEP) was assessed at 37 °C after 3-min-long incubation; the reaction was terminated by centrifugation (10,000 *g* for 1 min). The amount of product in the resulting supernatant was assessed by spectrometry at 240 nm. For positive control, human α-enolase (5 μg in 1 mL of reaction buffer) was incubated for 3 min with 2-PGA (6 mmol/L) and without substrate.

### In vitro study of interaction between the enolase-like surface protein and human plasminogen

Binding of human plasminogen by *P. aeruginosa* surface enolase-like protein was assessed by immunoblotting. After SDS-PAGE electrophoresis, the protein was transferred to a PVDF membrane. Next, the membrane was incubated with 10 μg of plasminogen in 2 mL of TBS at 37 °C for 1 h. Unbound plasminogen was washed off with TBS. A control assay was carried out with rabbit anti-human plasminogen antibodies instead of anti-alpha-enolase antibodies.

### Localization of enolase-like surface receptors of *P. aeruginosa* cells by electron microscopy

The *P. aeruginosa* cells (24-h-long culture in tryptic soy broth (TSB) liquid medium) were washed off thrice with phosphate-buffered saline (PBS) by centrifugation (5,000 *g* for 10 min at 4 °C). The pellet was blocked with 1 % BSA in 500 μL of PBS for 30 min at 25 °C. The precipitate of bacterial cells was subjected to immunocytochemical reaction with rabbit anti-alpha-enolase polyclonal antibodies with 1 % BSA for 12 h. Unbound antibodies were rinsed off with PBS by centrifugation (thrice at 5,000 *g* for 10 min at 4 °C); next, a marker was added—18-nm colloidal gold conjugated with goat anti-rabbit IgG polyclonal antibodies diluted 1:10 in PBS with 1 % of BSA. After a 3-h incubation at 25 °C, the excess of antibodies was washed off (thrice with PBS), and 10 % BSA was added to the pellet in 1:2 dilution for 2 h at 4 °C. Next, 10 % formalin was added in 1:10 dilution. Once incubated for 1 h at 4 °C, *P. aeruginosa* cells underwent dehydration in a series of alcohol/acetone baths. Once dehydrated and saturated with Epon 812 and DMP-30 catalyst, the sample was polymerized at 60 °C for 10 days. The blocks were sliced with an ultramicrotome, stained with toluidine blue and evaluated under JEOL 1011 transmission electron microscope.

### Electron microscopy assay of plasminogen binding by *P. aeruginosa* cells

*P. aeruginosa* cells (a 24-h culture in TSB liquid medium) were rinsed off thrice with PBS by centrifugation at 5,000 *g* for 10 min at 4 °C. The pellet was incubated with 30 μg of plasminogen in 250 μg PBS; next, the unbound protein was rinsed off thrice with PBS by centrifugation at 5,000 *g* for 10 min at 4 °C. Once blocked with 1 % BSA in 500 μL PBS for 30 min at 25 °C, the sample was washed off thrice with PBS by centrifugation at 5,000 *g* for 10 min at 4 °C. The sediment of bacterial cells underwent immunocytochemical reaction with rabbit anti-alpha-enolase polyclonal antibodies diluted 1:10 in PBS with 1 % BSA for 12 h. The unbound antibodies were washed off with PBS by centrifugation at 5,000 *g* for 10 min at 4 °C.

## Results

### Purification of the enolase-like surface protein from *P. aeruginosa* cells

**Scheme 1:**
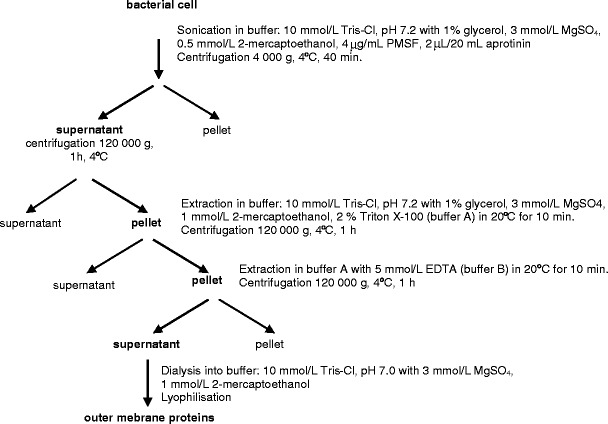
Isolation of outer membrane proteins from *Pseudomonas aeruginosa*

**Fig. 1:**
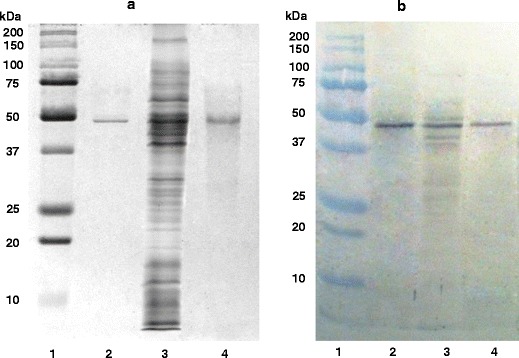
SDS-polyacrylamide gel electrophoresis (**a**) and immunoblot (**b**) of enolase-like protein. *1* molecular mass protein standards, *2* purified human α-enolase from the kidney (5 μg), *3* outer membrane proteins after extraction (150 μg), *4* enolase-like protein after preparative electrophoresis (10 μg)

The purification method of *P. aeruginosa* surface enolase-like protein involved the use of sonication, extraction, and preparative electrophoresis. The first step of isolating the surface proteins was sonication of bacteria suspended in a buffer containing a protease inhibitor. Next, the undisrupted cells were removed by centrifugation, and the resulting supernatant was ultracentrifuged with the aim to separate the cellular membrane fraction from the cytosolic one. The resulting sediment contained protein fractions bound to external and cytoplasmic membranes. After undergoing extraction cycles in detergent buffers containing a complexing compound (as in Scheme 1), a mixture of *P. aeruginosa* external membrane proteins was received (Fig. 1, row 3). Preparative electrophoresis for separation of membrane proteins and immunoblotting assay for detection allowed obtaining a purified membrane protein of electrophoretic mobility of 47 kDa and high reactivity with anti-alpha-enolase antibodies. Figure 1a shows the purification level after successive isolation stages, while Fig. 1b depicts the reaction between the purified protein and antibodies. Table 1 presents the protein purification balance.

**Table 1:** Purification result of enolase-like outer membrane protein from 18.7-g bacterial mass of *Pseudomonas aeruginosa*

	Steps	Protein mass (mg)	Yield (%)
1	Cell extract	6,350	100
2	EDTA extraction, dialysis, and lyophilization	547.8	8.62
3	Preparative electrophoresis	6.43	0.101

### Enzymatic activity of surface proteins of *P. aeruginosa*

Undisrupted cells of clinical strain resuspended in reactive buffer were used to determine the enzymatic activity of the bacterial enolase-like surface protein. Under these conditions, no cell lysis was observed. Following incubation of the bacteria with substrate (2-PGA), the absorbance was measured and compared with the control. The obtained results suggest that the enolase-like surface protein of *P. aeruginosa* does not exhibit enzymatic enolase activity (Table 2). Also, no enolase enzymatic activity was detected among the cytosolic fraction proteins.

**Table 2:** Enolase activity of outer membrane proteins of intact *Pseudomonas aeruginosa* cells

Dilution of intact bacterial cells	Control (*A*_240_)	Test (*A*_240_)	Δ*A*_240_
1:1	0.319 ± 0.014	0.325 ± 0.017	0.006
1:2	0.281 ± 0.012	0.283 ± 0.013	0.002
1:4	0.207 ± 0.022	0.216 ± 0.021	0.009
1:8	0.189 ± 0.019	0.199 ± 0.021	0.010
1:16	0.193 ± 0.016	0.194 ± 0.013	0.001
α-Enolase	0.089 ± 0.015	1.06 ± 0.051	0.967

### Interaction between the enolase-like surface protein and human plasminogen

**Fig. 2:**
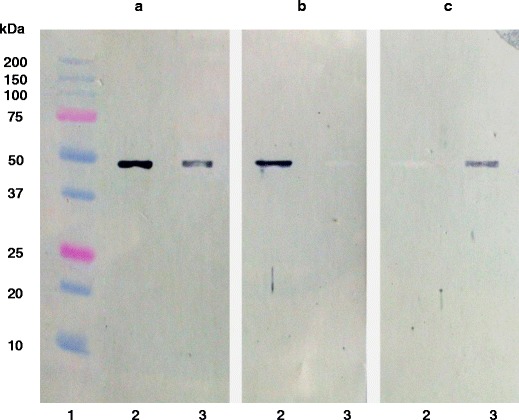
Interaction of enolase-like protein with plasminogen. *1* molecular mass protein standards, *2* purified human α-enolase (10 μg), *3* purified enolase-like protein (10 μg). **a** Immunoblot with anti-α-enolase antibodies. **b** Immunoblot with anti-α-enolase antibodies after reaction with plasminogen. **c** Immunoblot with anti-plasminogen antibodies after reaction with plasminogen

Immunoblotting assay allowed assessment of the plasminogen-binding capacity of the enolase-like membrane protein. Following electrophoresis, human alpha-enolase from the kidney and membrane enolase-like protein of *P. aeruginosa* were transferred to the Immobilon P membrane. Both proteins exhibited reactivity with rabbit anti-alpha-enolase polyclonal antibodies (Fig. 2a). The immunoblots, blocked with plasminogen and then treated with anti-alpha-enolase polyclonal antibodies, revealed that *P. aeruginosa* membrane enolase-like protein bound plasminogen and thus did not form complexes with antibodies (Fig. 2b, row 3); however, no such interaction has been observed in the case of alpha-enolase from the human kidney (Fig. 2b, row 2). These results were confirmed by an immunoblot incubated with plasminogen and subsequently developed with anti-plasminogen antibodies (Fig. 2c).

### Localization of enolase-like surface receptors of *P. aeruginosa* cells

**Fig. 3:**
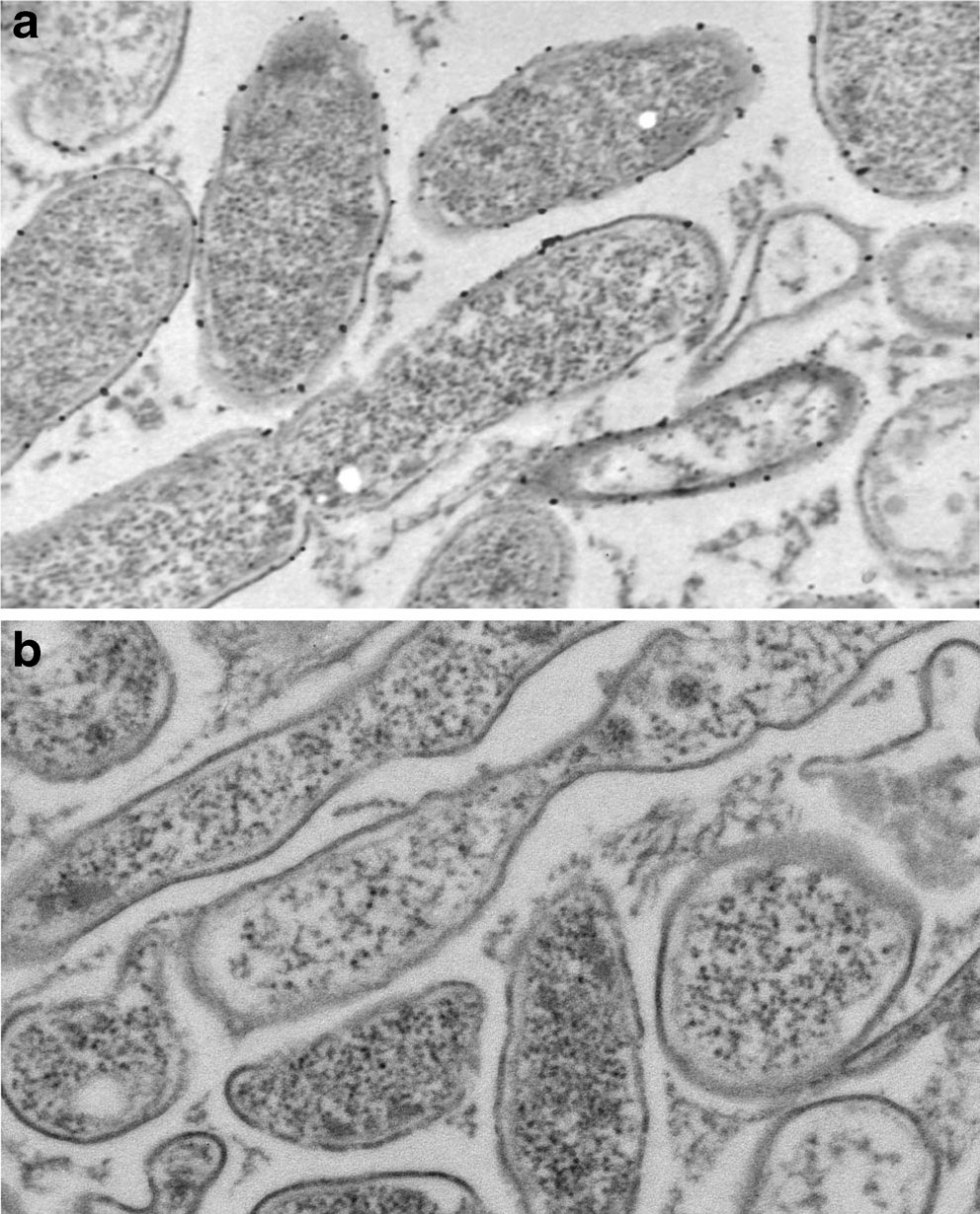
Electron microscopic localization of enolase-like protein on the surface of *Pseudomonas aeruginosa* cells using 18-ng gold particles (magnification × 60,000). **a** Reaction with anti-α-enolase antibodies. **b** Reaction with anti-α-enolase antibodies after adding plasminogen

Electron microscopy assay with the use of goat anti-rabbit IgG antibodies conjugated with 18-nm colloidal gold allowed localizing the enolase-like surface protein. The rabbit anti-human alpha-enolase polyclonal antibodies bind to the enolase-like protein on the bacterial surface (Fig. 3a). However, bacterial cells blocked with human plasminogen did not express any reaction with rabbit anti-human alpha-enolase polyclonal antibodies. No complex forming with antibodies conjugated with colloidal gold was observed in the sample (Fig. 3b).

## Discussion

The outer membrane is the first line of defense for Gram-negative bacteria against toxic compounds. The bacterial surface components are immunologically important proteins because of their accessibility to host defense reaction. The presence of antibodies against alpha-enolase has been reported to be associated with a diverse range of bacterial infections. Antibodies against human and bacteria enolases have been demonstrated to have cross-reactivity, suggesting that pathogen-related enolase may be involved in the development of autoimmune diseases (Liu and Shih 2007; Terrier et al. 2007). The alpha-enolase obtained from human tissue could be used as a specific antigen for revealing circulating pathologic antibodies; furthermore, the production of specific rabbit antibodies would allow the evaluation of possible cross-reactivity with *P. aeruginosa* surface enolase-like protein. This was the rationale for designing the method of obtaining alpha-enolase from the organ which contains the enzyme in high concentration, i.e., the human kidney. During evolution, the human enolase preserved its highly conservative structure, with the enzyme homology between the placental mammals and other organisms reaching 50 % (Piast et al. 2005; Seweryn et al. 2007). Therefore, some structural similarity could be anticipated between the human enolase and the enolase-like proteins expressed on the surface of *P. aeruginosa* external membranes, as described by Witkowska et al. (2005). In view of these studies, it seemed important to examine whether the enolase-like protein exhibited catalytic activity, despite the fact that *P. aeruginosa* is a non-fermenting bacteria using the Entner-Doudoroff pathway for oxidative catabolism. Our results have indicated that surface enolase-like protein does not have catalytic activity. This fact suggested that this protein has no catalytic center or that the center is unavailable to the substrate. In the present study, by electron microscopy, we have identified enolase-like protein on the surface of *P. aeruginosa*. We have confirmed the similarity of epitopes between the bacterial and human enolase. In fact, Bergmann et al. and Yavlovich et al. have identified alpha-enolase on the surface of Gram-positive bacteria such as *Streptococcus pneumoniae*, *Streptococcus pyogenes*, *Staphylococcus aureus*, and *Mycoplasma fermentans* (Bergmann et al. 2001; Yavlovich et al. 2007; Diaz-Ramos et al. 2012). We have identified for the first time on the surface of *P. aeruginosa* (Gram-negative bacteria) enolase-like protein. Moreover, we have obtained and purified this protein and described it. The study of localizing the enolase-like protein has been carried out on the surface of undisrupted bacterial cells by using antibodies against human alpha-enolase. The binding of enolase-like protein to plasminogen was demonstrated by electron microscopy in an in vitro study. Interestingly, the bacterial cells that have incubated with plasminogen did not exhibit any interaction with antibodies against human alpha-enolase. This result confirmed that the enolase-like protein on the surface of *P. aeruginosa* possesses the ability to bind to plasminogen. It was shown previously that surface proteins exhibit receptor-like properties in *S. pneumoniae*, *Streptococcus mutans*, *Trichomonas vaginalis*, and *Bacillus anthracis* (Pancholi and Fischetti 1998; Bergmann et al. 2001; Whiting et al. 2002; Ge et al. 2004; Agarwal et al. 2008; Mundodi et al. 2008). Plasminogen, in turn, allows it to invade tissues contributing to its pathogenicity. Proteolytic activation of plasminogen results in the formation of plasmin that degrades fibrin and ECM proteins (Diaz-Ramos et al. 2012). In conclusion, we have demonstrated for the first time, to our knowledge, that *P. aeruginosa* enolase-like protein has affinity for human plasminogen. Our results indicate that the surface enolase of a bacterium could be a key molecule in pathogenesis by facilitating bacterial interaction with host cells. Binding of plasminogen via enolase-like surface protein to the surface of *P. aeruginosa* may therefore facilitate invasion of host tissue.
